# Registered Dietitians' Knowledge, Attitudes and Use of Simulation‐Based Education in Ireland: A Mixed‐Methods Study

**DOI:** 10.1111/jhn.70326

**Published:** 2026-07-29

**Authors:** Aoibheann Power, Clodagh Lee, Clare Corish, Annelie Shaw

**Affiliations:** ^1^ University College Dublin, School of Public Health, Physiotherapy and Sports Science Dublin Ireland

**Keywords:** dietitian, mixed‐methods, pre‐registration training, simulation‐based education

## Abstract

**Introduction:**

Simulation‐based education (SBE) is increasingly used in healthcare training to support the development of communication, clinical reasoning and professional competence. Despite its growing use in medicine and nursing, little is known about how SBE is understood and used within dietetics in Ireland. This study investigated dietitians' knowledge, attitudes and use of SBE, and explored factors influencing its implementation in pre‐registration dietetic education.

**Methods:**

A convergent mixed‐methods design was used. An online questionnaire was distributed to CORU‐registered dietitians in Ireland, assessing familiarity with SBE, attitudes, use, and perceived barriers and facilitators. Data were analysed using descriptive and inferential statistics. Semi‐structured interviews were conducted with a sample of respondents and analysed using thematic analysis informed by the Capability, Opportunity, Motivation‐Behaviour (COM‐B) model. Quantitative and qualitative findings were integrated at interpretation using the COM‐B framework.

**Results:**

The study included 114 dietitians, of whom 15 participated in the qualitative component. Although 78% had been exposed to simulation activities and 85% expressed interest in further training, only 14% of respondents reported using SBE. Greater familiarity with SBE was associated with more positive attitudes, stronger motivation, and fewer perceived resource and cost barriers (*p* < 0.001). Years of professional experience was the only significant predictor of SBE use. Qualitative findings revealed uncertainty about what constitutes SBE, with many dietitians using role‐play or case‐based activities without identifying them as simulation. Participants viewed SBE as a supportive tool, but not a replacement for real patient contact. Key barriers included limited time, educator training, resource constraints and limited institutional support. Enablers included shared resources, interdisciplinary collaboration and clearer guidance from higher education institutions.

**Conclusion:**

Dietitians in Ireland support SBE, but its use remains inconsistent due to gaps in understanding. Coordinated educator training, clearer definitions and stronger institutional support may enable more effective integration of SBE into dietetic education.

## Summary

- Registered dietitians in Ireland recognise the educational value of simulation‐based education (SBE) for building confidence, communication, and clinical skills, but current implementation in pre‐registration training is inconsistent and largely informal.
- Poor understanding of what constitutes SBE, lack of formal training, limited institutional support, and time constraints restrict uptake, despite high motivation among educators.
- Enhancing SBE use requires clearer definitions and clarity on activities that constitute simulation, targeted educator training, shared resources, and institutional support to embed SBE effectively as a preparatory and supportive strategy in pre‐registration dietetic education.

## 1 Introduction

Simulation‐based education (SBE) is an experiential learning approach that enables learners to practise clinical, communication and professional skills in a safe and controlled environment [1]. It spans a continuum from low‐fidelity methods such as role play and case‐based scenarios to high‐fidelity simulations using standardised patients or immersive technologies [1]. Across health professions, SBE is widely recognised for its capacity to bridge the gap between theoretical learning and real‐world clinical practice by supporting skill development, decision‐making and reflective learning [2]. Within medicine, nursing and allied health professions, SBE has been shown to improve clinical reasoning, communication, teamwork and confidence, while also enhancing preparedness for practice and patient safety [3, 4, 5].

In dietetics, elements of simulation have long been embedded within education and training, including role‐play, case‐based discussions, objective structured clinical examinations (OSCEs) and standardised patient encounters [6]. However, the explicit framing of these activities as SBE, and their systematic integration into curricula and practice‐based education, remains inconsistent. Dietetic education is guided by professional competency or proficiency frameworks, including those set out by the Health and Social Care Professionals Council (CORU) in Ireland, which emphasise the importance of communication, collaboration, professional autonomy, accountability, safety and quality, professional development and professional knowledge and skills [7]. SBE aligns closely with these proficiencies by providing opportunities for learners to practise complex consultations, apply the nutrition care process and develop interprofessional skills within a safe learning environment before engaging with real patients. Although established simulation best practice standards exist across healthcare to support the design, delivery and evaluation of simulation [1], the terminology used to describe simulation within dietetics remains inconsistent. Activities such as role‐play or standardised patient encounters are not always explicitly identified as simulation‐based education. This inconsistency may limit shared understanding, hinder comparisons across studies and programmes, and make it more difficult to synthesise evidence and identify best practice within the profession. Furthermore, there is currently no profession‐specific framework describing how simulation should be best implemented within dietetic education.

Internationally, a growing body of literature demonstrates that SBE enhances dietetic students' confidence, communication skills, self‐efficacy and readiness for clinical placement. Studies across a range of countries have reported that simulated patient encounters, OSCEs, interprofessional simulations and virtual or tele‐simulation approaches improve students' perceived competence and support the development of professional behaviours [8, 9, 10, 11, 12, 13, 14, 15, 16, 17, 18, 19, 20, 21, 22, 23, 24, 25, 26, 27, 28, 29, 30, 31, 32, 33, 34, 35, 36]. Systematic and narrative reviews further suggest that SBE can improve both perceived and observed clinical competence in dietetic students [37, 38]. However, it should be noted that much of the available evidence is based on short‐term educational outcomes, with relatively limited evidence demonstrating sustained transfer to clinical practice or patient outcomes. Furthermore, most existing research focuses on student outcomes, with relatively little attention given to the perspectives of dietetic educators and clinical supervisors who are responsible for implementing and sustaining SBE within dietetic programmes.

Educator perspectives are critical to the successful integration of SBE. Studies from Australia, Saudi Arabia and the United States suggest that dietetic educators and preceptors generally hold positive views of simulation but face substantial barriers to implementation, including limited time, lack of training, insufficient access to facilities and uncertainty about how simulation should be used in practice‐based education [39, 40, 41, 42]. Many educators express a preference for real patient experience and report concerns about the feasibility and resource demands of simulation, particularly in clinical placement settings [43]. These challenges highlight the importance of understanding not only whether SBE is valued, but also how it is interpreted, operationalised and supported within the profession.

Within Ireland, there is increasing institutional and policy interest in simulation across healthcare education, including the development of national simulation frameworks and university‐based initiatives supporting interprofessional and technology‐assisted learning [44, 45]. However, no published studies have examined how registered dietitians in Ireland understand, value or use SBE in their roles as educators, supervisors and practitioners. Without this evidence, it is difficult for higher education institutions, clinical placement providers and professional bodies to design training, allocate resources or develop guidance that reflects the realities of dietetic practice.

Implementation of SBE within dietetics is not solely a matter of access to technology or facilities. Behaviour change theory suggests that adoption of educational innovations is influenced by individuals' capability, opportunity and motivation to engage in the desired behaviour. The Capability, Opportunity, Motivation–Behaviour (COM‐B) model [46] provides a useful framework for examining these factors in relation to educators' use of SBE. Capability includes knowledge and skills to design and facilitate simulation; opportunity encompasses environmental and organisational factors such as time, space, resources and institutional support; and motivation reflects beliefs, attitudes and professional identity. Applying the COM‐B model allows for a theoretically informed understanding of why SBE may be underused despite positive attitudes toward its educational value.

This study aimed to investigate registered dietitians' knowledge, attitudes and use of SBE in Ireland, and to explore the factors influencing its implementation in pre‐registration dietetic education. A convergent mixed‐methods design was used, combining a national survey with in‐depth qualitative interviews informed by the COM‐B framework. By integrating quantitative and qualitative data, this study sought to provide a comprehensive understanding of how SBE is currently understood and utilised within the Irish dietetic profession, and to identify practical and theoretical implications for future education and workforce development.

## 2 Materials and Methods

### 2.1 Study Design

A convergent mixed‐methods design was used to examine registered dietitians' knowledge, attitudes and use of SBE in Ireland and to explore factors influencing its implementation in dietetic education. Quantitative and qualitative data were collected within the same study period and analysed separately before being integrated during interpretation, using the COM‐B framework as a common analytic lens. Survey results identified patterns in knowledge, attitudes and use of SBE across the profession, while interview data provided contextualised explanations, enabling triangulation and a more comprehensive understanding of barriers and enablers to implementation.

The quantitative component comprised an online survey of CORU‐registered dietitians, while the qualitative component involved semi‐structured interviews with a sample of survey respondents. Participants either volunteered to participate following the survey or were directly invited to ensure variation in experience and practice setting. The qualitative interview schedule and analysis were informed by the Capability, Opportunity, Motivation‐Behaviour (COM‐B) framework to enable a theory‐based exploration of factors influencing SBE implementation.

The research team included two MSc dietetics students who conducted the interviews and analysis, and two academic supervisors with expertise in dietetics and simulation‐based education. Reflexive discussions and supervisory oversight were used to mitigate potential bias arising from researchers' own experiences of SBE. The use of a pre‐defined interview guide and a consistent coding approach across all transcripts further helped to minimise the influence of the researchers' positionality during the qualitative data collection and analysis.

### 2.2 Participants and Recruitment

Eligible participants were CORU‐registered dietitians currently practising in Ireland in any area of practice, including clinical, community, academic, private and industry settings. Dietitians who were not actively practising or not registered with CORU were excluded.

For the survey, recruitment was undertaken using a multi‐channel snowball sampling strategy to maximise reach across the profession. Invitations were circulated via dietetic managers in hospital and community services, the Irish Nutrition and Dietetic Institute (INDI) weekly e‐zine, practice placement networks, an institutional MSc alumni mailing list, and professional social media channels. Participants were encouraged to forward the survey link to colleagues. Approximately 205 invitations were distributed directly by email, with additional indirect reach via forwarded links and professional networks.

Study information circulated during recruitment included details of both the survey and the qualitative interview component. Dietitians were invited to participate in the survey and a semi‐structured interview. All survey respondents who indicated willingness to be interviewed before the survey closed were invited to participate in the qualitative phase.

### 2.3 Quantitative Data Collection and Analysis

An anonymous online questionnaire was developed and hosted on an institutional GDPR‐compliant SurveyMonkey enterprise platform. Informed consent was obtained via an electronic tick‐box at the start of the survey, and no identifying information was collected. Prior to commencing the questionnaire, participants were provided with a participant information sheet, which was also embedded at the beginning of the survey. This included a brief explanation of SBE, together with examples of different simulation activities. Participants also received a link to an animated participant information video that reinforced the explanation of SBE.

The questionnaire was informed by previously validated Knowledge–Attitudes–Practice instruments used in other healthcare professions and adapted for the Irish dietetic context. Items were also informed by the structure of the KAP tool developed by Jin, Lu, and Pang for nursing educators [47]. The final instrument comprised four sections: participant characteristics, knowledge and familiarity with SBE, attitudes toward SBE, and current use, barriers and facilitators. Likert‐scale items were scored on five‐point scales (from strongly disagree to strongly agree), with selected items reverse‐scored to minimise response bias. The survey was designed to capture participants' perspectives on SBE across both university‐based dietetic education and practice‐based training of pre‐registration dietetic students. Most questions explored participants' views of SBE broadly, rather than focusing on specific individual simulation modalities. The questionnaire was piloted with three registered dietitians, resulting in minor wording and flow refinements.

Data collection was open from 30th July to 30th September 2025. A total of 134 responses were recorded. Responses were excluded if more than 50% of items were missing or if key outcome variables were not completed. Duplicate entries were identified through timestamps and demographic patterns, with the earliest retained. After data cleaning, 114 valid responses were included in the final analysis.

Survey data were analysed using IBM SPSS Statistics (version 29). Categorical variables were summarised as frequencies and percentages, and ordinal or scale variables as medians and interquartile ranges. Associations between categorical variables were examined using chi‐square tests or Fisher's exact tests where appropriate, with effect sizes reported using Cramer's V. Relationships between ordinal variables were explored using Spearman's rank‐order correlations, and Kendall's tau was used for associations involving binary variables.

Binary logistic regression was conducted to identify predictors of two outcomes: current use of SBE and interest in further SBE training. Predictor variables included familiarity with SBE, prior exposure, attitudes toward effectiveness, perceived workload and resource burden, motivation, professional role, and years of experience. Multicollinearity and model assumptions were checked prior to analysis, and odds ratios with 95% confidence intervals were reported. Statistical significance was set at *p* < 0.05.

### 2.4 Qualitative Data Collection and Analysis

Semi‐structured interviews were conducted online using Zoom® by a trained researcher, with a second researcher present to record field notes on non‐verbal cues and contextual factors. Before each interview, participants were given a brief introduction to SBE and were given the opportunity to ask questions or seek clarification on the topic if needed. Interviews lasted between 25 and 45 min and explored participants' experiences of SBE, perceptions of its role in university and practice‐based education, and perceived barriers and enablers to implementation. The interview guide was developed using the five‐step process for constructing semi‐structured interview schedules recommended by Kallio et al. [48] and was organised around the COM‐B domains of capability, opportunity and motivation.

Interviews were audio‐ and video‐recorded with consent and transcribed verbatim. Automated transcripts were checked against recordings and corrected for accuracy, with all identifying information removed. Two pilot interviews were conducted to test question clarity and flow and were retained in the final analysis as no substantive changes were required [49, 50]. Data collection continued until 15 interviews were completed. Code saturation was reached by interview eight, with no new codes identified after this point [51]. However, all remaining scheduled interviews were completed to support meaning saturation and deepen understanding of existing themes [52].

Interview transcripts and field notes were imported into NVivo® (version 20) for analysis. Thematic analysis was conducted using a combined deductive and inductive approach. Initially, transcripts were coded deductively according to the COM‐B domains of capability, opportunity and motivation. Inductive coding was then undertaken to identify additional themes not captured by the COM‐B framework. Themes and subthemes were reviewed and refined through discussion between the two researchers until consensus was reached.

### 2.5 Ethics

The study was classified as low risk, as no sensitive or identifying data were collected. Ethical approval was granted by the University College Dublin Human Research Ethics Committee (UTMREC‐25‐36‐LEE‐POWER‐SHAW). All participants provided informed consent and were assured of confidentiality and the voluntary nature of participation.

## 3 Results

### 3.1 Participant Characteristics

A total of 114 CORU‐registered dietitians completed the survey, representing approximately 8% of the dietetic workforce in Ireland. Most respondents were working in clinical roles (63%), followed by community (24%), academic (4%), private practice or freelance (4%), industry (3%) and management roles (3%). Approximately half were educated to MSc level and over one third had more than 16 years of professional experience. Only 9.6% reported having received formal training in SBE, while 68.5% had reported receiving no training. Further details of the participant characteristics can be found in Table 1.

**Table 1:** Participant characteristics of CORU‐registered dietitians in Ireland responding to a survey on knowledge, attitudes, and use of simulation‐based education (SBE).

Participant characteristic (*n* = 114)	*n* (%)^a^
Education	
BSc	52 (45.6)
MSc	58 (50.9)
Doctoral	4 (3.5)
Current role	
Clinical	72 (63.2)
Community	27 (23.7)
Academic	5 (4.4)
Industry	3 (2.6)
Private Practice/Freelance	4 (3.5)
Manager	3 (2.6)
Experience (y)	
0–5	37 (32.5)
6–15	35 (30.7)
16+	42 (36.8)
Level of SBE training	
Formal	11 (9.6)
Informal	25 (21.9)
None	78 (68.5)

Abbreviations: SBE, simulation‐based education; y, years.

^a^ Values reported as frequencies (*n*) and percentages (%).

Fifteen dietitians participated in semi‐structured interviews. Participants were predominantly female (93%), with a median of 19 years' experience, and represented university, acute, community and private practice settings. Although 67% had used some form of SBE with students, only 40% were familiar with the term SBE prior to the study, and just one third had received any form of SBE training.

### 3.2 Capability: Knowledge and Understanding of Simulation‐Based Education

Survey data indicated low to moderate familiarity with SBE. Only 27% of respondents rated themselves as moderately or extremely familiar, while 73% reported little or no familiarity. Despite this, 78% reported having used or been exposed to some form of SBE during training or practice, most commonly role‐play and case‐based discussions. Exposure to higher‐fidelity modalities, such as standardised patients, interprofessional simulation or virtual reality, was much lower. Details of familiarity with and use of SBE can be found in Table 2.

**Table 2:** Self‐reported familiarity with and use of simulation‐based education (SBE) among CORU‐registered dietitians in Ireland.

Familiarity towards SBE	*n* (%)^a^
Not at all	17 (14.9)
Slightly/somewhat	66 (57.9)
Moderately	25 (21.9)
Extremely	6 (5.3)

SBE activity	*n* (%)^b^
Role play	94 (82.5)
Case‐based discussions	85 (74.6)
OSCE	58 (50.9)
Interprofessional simulation scenarios	28 (24.6)
Standardised patients	25 (21.9)
High‐fidelity mannequins	18 (15.8)
Virtual reality simulation	8 (7.0)

Abbreviations: OSCE, Objective Structured Clinical Examination; SBE, simulation‐based education.

^a^ Familiarity was assessed using a 4‐point Likert scale ranging from 1 (“not at all”) to 5 (“extremely”). Values represent the number and percentage of participants within each category.

^b^ Multiple responses were permitted. Values represent the *n* and (%) of participants selecting each SBE activity.

Interview data provided an important distinction between familiarity with the terminology surrounding simulation and experience of simulation activities. While 60% of interview participants reported that they had not been familiar with the term SBE or what it encompassed prior to participating in the study, 93% described previous experience with simulation activities. Many participants initially associated SBE with high‐technology or high‐fidelity approaches and did not recognise lower‐fidelity activities, such as role‐play or scenario‐based discussions, as simulation. Several described having used simulation‐type activities for years without labelling them as such.

I would have thought it was something a bit more high‐tech…

(P10)

We've probably always been doing it, but never called it that…

(P7)

Even participants who were confident using simulation often expressed uncertainty about whether what they were doing “counted” as SBE, while some who felt confident demonstrated limited conceptual understanding. This mismatch suggests that knowledge gaps and inconsistent terminology may contribute to under‐recognition and under‐reporting of SBE use.

Greater familiarity with SBE was strongly associated with more positive beliefs about its educational value, including perceptions of effectiveness, contribution to communication skills, learner engagement and critical thinking (*p* < 0.001). Familiarity was also associated with greater motivation to use SBE and fewer concerns about cost and resource demands (*p* < 0.001).

### 3.3 Opportunity: Settings, Resources and Institutional Support

From the survey, the most commonly reported barriers to SBE were lack of knowledge or training (59%), lack of awareness of available resources (58%), limited time and resources (47%), and limited institutional support (37%). Technology cost and perceived irrelevance were reported much less frequently. Details of perceived barriers and facilitators can be found in Table 3.

**Table 3:** Perceived barriers and facilitators to the integration of simulation‐based education (SBE) within dietetic curricula among CORU‐registered dietitians in Ireland.

	*n* (%)^a^
Barriers	
Lack of knowledge/training	67 (58.8)
Lack of awareness of available resources	66 (57.9)
Limited time/resources	54 (47.4)
Limited institutional support	42 (36.8)
Lack of confidence in facilitating SBE	39 (34.2)
High technology cost	23 (20.2)
Perceived lack of relevance	13 (11.4)
Facilitators	
Better knowledge/understanding of SBE	106 (93.0)
Informal training	91 (80.5)
Access to simulation resources	89 (78.1)
Exposure to SBE	74 (64.9)
Institutional/managerial support	71 (62.3)
Formal training	46 (40.4)

^a^ Multiple responses were permitted. Values represent the number and percentage of respondents selecting each barrier and facilitator.

Abbreviation: SBE, simulation‐based education.

Interview participants consistently emphasised opportunity‐related constraints. Time pressures, competing clinical workloads, limited physical space and lack of access to suitable materials or scenarios were perceived as major obstacles, particularly within practice placement settings. Several participants noted that even when they were motivated to use SBE, the absence of institutional structures, such as protected educator time, training or ready‐to‐use resources, made implementation difficult.

Well, it's very time‐consuming…

(P1)

We're never given opportunities to understand or learn more about it or to do training in it.

(P15)

…if there were scenarios set up and ready to go…I think those kind of resources would be brilliant…

(P9)

There was strong support for SBE within university‐based education, particularly as preparation for practice placements. Views on SBE in placement settings were more nuanced. Many participants felt SBE could be useful if embedded informally, e.g. through short role‐plays or case discussions before or after patient consultations, but some expressed concern that simulation should not displace real patient contact, given the experiential nature of dietetic practice.

I think it's useful but I don't think you can use it completely…I would never like to see it used to replace the real‐life experience of placement.

(P1)

Across both datasets, institutional and managerial support emerged as a key enabler. Over 60% of survey respondents selected institutional support as a facilitator, and interviewees highlighted the importance of guidance from higher education institutions, access to educator training and interdisciplinary sharing of resources.

### 3.4 Motivation: Attitudes, Confidence and Professional Values

Attitudes toward SBE were overwhelmingly positive. Over 85% of survey respondents agreed that SBE is an effective teaching strategy, enhances communication skills and adds value to real‐world experience. Most agreed that SBE is more engaging than traditional teaching and better supports critical thinking. The majority (65%) also believed that SBE is currently underutilised in dietetic training.

Motivation was high, with 85% expressing interest in further SBE training and over two‐thirds indicating willingness to spend more time using SBE with students. Higher motivation was strongly associated with interest in training (*p* < 0.001).

Interview participants echoed these findings, describing SBE as a valuable way to build student confidence, prepare learners for challenging consultations and support reflective practice. Many reflected on their own experiences of feeling nervous or uncomfortable during simulations yet acknowledged their value in developing professional competence. A small number expressed concerns that poorly designed SBE could undermine confidence or create unrealistic expectations, and some cautioned that over‐reliance on simulation might inadequately prepare students for the complexity of real patients.

…one thing students find the hardest is to gain that confidence on the wards and with patients… if you get more comfortable with that before you see an actual patient, it'll come easier for you on the ward.

(P4)

…one of the values is it increases not only knowledge, but it increases confidence, for the students, and you don't want it to have the opposite effect…maybe if it's ill‐prepared it will have the opposite effect.

(P12)

Importantly, both datasets highlighted that positive attitudes alone were insufficient to drive behaviour. Years of professional experience was the only significant predictor of current SBE use (*p* = 0.027), suggesting that more experienced dietitians may have greater confidence or autonomy to adopt new educational approaches.

Additional illustrative interview quotations supporting each theme are provided in Supplementary Table 1. Additional survey findings related to dietitians' attitudes towards SBE are presented in Supplementary Table 2.

### 3.5 Integration of Findings

Together, the quantitative and qualitative findings show that while Irish dietitians are highly motivated and supportive of SBE, its implementation is constrained by limited capability and opportunity. Uncertainty about what constitutes SBE, lack of educator training, and insufficient institutional infrastructure restrict the translation of positive attitudes into practice. Where SBE is used, it is most often low‐fidelity and informal, embedded within existing teaching practices rather than delivered as structured simulation programmes.

## 4 Discussion

This mixed‐methods study provides the first insight into registered dietitians' knowledge, attitudes and use of SBE in Ireland. Although most dietitians reported positive attitudes towards SBE and strong interest in further training, current use remains low. By integrating survey data with in‐depth COM‐B informed interviews, this study demonstrates that the limited uptake of SBE is not due to lack of perceived value, but rather to constraints in capability and opportunity.

An important finding was the disconnect between reported experience with SBE and familiarity with the term itself. While most respondents had engaged in activities such as role‐play, case‐based discussions and OSCEs, fewer than one third described themselves as familiar with SBE. Interview data confirmed that many educators did not conceptualise these activities as simulation, instead associating SBE primarily with high‐fidelity or technology‐driven approaches. Similar misconceptions have been reported in other healthcare professions such as nursing and medicine, where simulation is often narrowly defined, limiting recognition of the full spectrum of pedagogically valid approaches [53, 54]. In dietetics specifically, terminology surrounding experiential learning, competency‐based education and simulation is often used interchangeably in the literature, with overlapping concepts and activities described using different terms. This lack of consistency can make it difficult to compare findings across studies and highlights the need for clearer conceptual definitions within dietetics and healthcare education. This lack of shared understanding may contribute to under‐recognition of existing good practice and hinder the coordinated development and consistent integration of simulation within dietetics curricula. Although established simulation standards provide guidance for the design, delivery and evaluation of simulation across healthcare professions, our findings suggest that greater awareness of these frameworks and their application within dietetics may support more consistent implementation and reporting of SBE.

Importantly, participants' lower self‐reported familiarity with SBE likely reflected their prior familiarity with the terminology rather than their understanding during data collection. Before completing the survey or interviews, participants were provided with an explanation of SBE and examples of simulation activities, allowing them to reflect on their experiences within a shared conceptual framework. Many participants subsequently recognised that they had previous experience with simulation activities but had not previously identified these as forms of SBE. Consequently, we believe the attitudes and perceptions reported in this study represent informed views of SBE rather than responses based on an incomplete understanding of the concept.

Attitudes toward SBE were strongly positive in this study. Survey respondents consistently endorsed its educational value, particularly for enhancing communication skills, critical thinking and learner engagement. These findings align with international evidence showing that SBE improves confidence, self‐efficacy and preparedness for practice among dietetic students [8, 9, 10, 11, 12, 13, 14, 15, 16, 17, 18, 19, 20, 21, 22, 23, 24, 25, 26, 27, 28, 29, 30, 31, 32, 33, 34, 35, 36]. Interview participants reinforced these benefits, particularly in relation to building confidence and preparing students for challenging patient interactions. Importantly, participants viewed SBE as supportive of, rather than a replacement for, real patient experience, aligning with similar perspectives reported by dietetic educators in Australia and the United States [40, 42]. While some evidence from nursing and allied health professions suggests that simulation can substitute for a portion of placement hours without compromising competence, the evidence base within dietetics remains limited [55, 56]. Given the communication‐focused nature of dietetic practice and the relatively short duration of dietetic placements, SBE may be better positioned as a preparatory and supportive learning strategy rather than a substitute for clinical experience.

Opportunity‐related barriers were prominent across both datasets. Lack of time, limited access to resources and insufficient institutional support were consistently identified as constraints, particularly in clinical placement environments. These findings mirror those of previous studies in which educators reported that workload pressures, lack of facilities and limited training impede simulation delivery [39, 40, 41, 42]. While technology cost is often cited as a barrier to simulation [43], it was relatively unimportant in this study, likely reflecting the predominance of low‐fidelity approaches such as role‐play and case‐based discussion in current dietetic practice. This highlights the potential for SBE to be implemented in pragmatic, low‐resource ways if educators are supported through training and institutional provision.

Motivation to engage with SBE was high, with most respondents expressing interest in further training and willingness to invest time in simulation activities. However, positive attitudes alone did not translate into reported behaviour, as years of professional experience was the only predictor of current SBE use. This finding is consistent with behaviour change theory, which proposes that capability and opportunity must be present alongside motivation for behaviour to occur [46]. The COM‐B framework was therefore helpful in explaining why enthusiasm for SBE does not necessarily result in widespread implementation.

The findings have important implications for dietetic education in Ireland. First, clearer definitions and shared language around SBE are needed to help educators recognise existing practice and identify opportunities for development. Second, targeted educator training is essential to build capability and confidence in designing, facilitating and debriefing simulation activities. Such training should be informed by established simulation standards and best‐practice frameworks [1, 45], which emphasise the importance of educator preparation, learner‐centred facilitation, psychological safety, effective pre‐briefing and structured debriefing. Adapting these principles to the context of dietetic education may facilitate more consistent implementation of SBE across university and practice settings. Third, institutional support, including access to shared resources, protected educator time and interprofessional collaboration, is critical to create the opportunity for SBE to be used consistently across university and placement settings. Future research should explore how established simulation standards can be translated into profession‐specific guidance that supports the consistent design, implementation and evaluation of SBE within dietetic education.

### 4.1 Strengths and Limitations

This study has several strengths including the use of a theory‐informed mixed‐methods design and the integration of quantitative and qualitative data. However, limitations should be acknowledged. The survey used snowball sampling, which may have introduced self‐selection bias, and the proportion of the national workforce represented was modest. The qualitative sample, while diverse in experience and setting, was relatively small. Furthermore, many participants in the qualitative sample were known to members of the research team through professional networks, introducing the potential for bias and social desirability effects [57]. While steps were taken to mitigate this, including the use of the COM‐B model to provide a structured and consistent analytical framework, the influence of these relationships cannot be fully excluded. Nonetheless, data saturation was achieved, and the integration of quantitative and qualitative datasets through triangulation strengthens confidence in the overall conclusions. An additional limitation relates to the measurement tool. While questionnaire development was informed by previously validated Knowledge–Attitudes–Practice instruments used in other healthcare professions, the specific questionnaire used in this study was not validated. Furthermore, the familiarity item was broad and may have been interpreted differently by participants, for example, as familiarity with the terminology, educational approach, or implementation of SBE, and should therefore be interpreted with caution.

## 5 Conclusion

Irish dietitians strongly support the educational value of SBE, yet its use in pre‐registration training remains inconsistent. This study shows that limited understanding of what constitutes simulation, alongside constraints related to time, training and institutional support, restricts implementation despite high motivation. Addressing these barriers through educator development, clearer guidance and coordinated national strategies may enable SBE to be more effectively embedded within dietetic education, supporting the preparation of future practitioners for contemporary clinical practice.

## Author Contributions

All listed authors made substantial contributions to the manuscript and have agreed to the final submitted version. Aoibheann Power was responsible for the qualitative component of the project, including data collection, analysis, and manuscript preparation. Clodagh Lee was responsible for the quantitative component of the project, including data collection, analysis, and manuscript preparation. Annelie Shaw conceived and designed the overall project and developed the survey and interview guide. Both Annelie Shaw and Clare Corish provided supervisory oversight on all aspects of the project and made significant contributions to the writing and revision of the manuscript.

## Funding

The authors have nothing to report.

## Conflicts of Interest

The authors declare no conflicts of interest.

## Supporting information


Supporting File 1

Supporting File 2

## Data Availability

The data that support the findings of this study are available in the Supporting material of this article.
