# Supplementary material for: Registered Dietitians' Knowledge, Attitudes and Use of Simulation‐Based Education in Ireland: A Mixed‐Methods Study
Source: J Hum Nutr Diet. 2026 Jul 29;39(4):e70326. doi: 10.1111/jhn.70326 (PMC13420780; doi:10.1111/jhn.70326)
Supplement: Supplementary file 1 — Supporting File 1 [file JHN-39-0-s002.docx]

***Supplementary table 2: Dietitians’ attitudes towards SBE for pre-registration training from national survey data in Ireland (n=114)***

| Item  *n* (%)^a^ | Strongly Disagree | Disagree | Neither agree/disagree | Agree | Strongly  Agree | Median (IQR) **^b^** |
| --- | --- | --- | --- | --- | --- | --- |
| **General effectiveness of simulation** | | | | | | |
| SBE is an effective teaching strategy. | 2 (1.8) | 1 (0.9) | 13 (11.4) | 67 (58.8) | 31 (27.2) | 4 (4.0-5.0) |
| SBE improves communication skills of pre-registered dietitians. | 0 (0) | 1 (0.9) | 14 (12.3) | 69 (60.5) | 30 (26.3) | 4 (4.0-5.0) |
| SBE is a valuable addition to real-world experience. | 0 (0) | 0 (0) | 10 (8.8) | 65 (57.0) | 39 (34.2) | 4 (4.0-5.0) |
| **Comparison to traditional teaching methods** | | | | | | |
| SBE can stimulate learners’ interest in learning. | 0 (0) | 2 (1.8) | 15 (13.2) | 58 (50.9) | 39 (34.2) | 4 (4.0-5.0) |
| SBE can better foster learners’ critical thinking. | 0 (0) | 1 (0.9) | 16 (14.9) | 58 (65.8) | 39 (34.2) | 4 (4.0-5.0) |
| **Workload and resource considerations** | | | | | | |
| SBE adds to my workload. | 3 (2.6) | 20 (17.5) | 57 (50.0) | 32 (28.1) | 2 (1.8) | 3 (3.0-4.0) |
| SBE is too expensive for dietetics. | 13 (11.4) | 48 (42.1) | 48 (42.1) | 4 (3.5) | 1 (0.9) | 2 (2.0-3.0) |
| SBE is too resource-intensive for dietetics. | 12 (10.5) | 54 (47.4) | 40 (35.1) | 7 (6.1) | 1 (0.9) | 2 (2.0-3.0) |
| **Personal interest and motivation** | | | | | | |
| Interested in SBE | 1 (0.9) | 2 (1.8) | 14 (12.3) | 71 (62.3) | 26 (22.8) | 4 (3.0-4.0) |
| Spend more time on SBE with pre-registration dietitians | 3 (2.6) | 10 (8.8) | 25 (21.9) | 57 (50.0) | 19 (16.7) | 4 (3.0-4.0) |
| Willing to work hard to overcome the difficulties encountered in SBE in practice. | 2 (1.8) | 7 (6.1) | 29 (25.4) | 61 (53.5) | 15 (13.2) | 4 (3.0-4.0) |
| **Beliefs about implementation** | | | | | | |
| Support the integration of SBE into practice placement education of pre-registration dietetic students | 1 (1.8) | 3 (2.6) | 14 (12.3) | 60 (52.6) | 35 (30.7) | 4 (4.0-5.0) |
| SBE should be facilitated by trained persons. | 0 (0) | 0 (0) | 9 (7.9) | 58 (50.9) | 47 (41.2) | 4 (4.0-5.0) |
| A high-fidelity simulator (e.g., mannequins) are essential for effective SBE | 4 (3.5) | 35 (30.7) | 45 (39.5) | 20 (17.5) | 10 (8.8) | 3 (2.0-4.0) |
| SBE is underutilised in dietetics training. | 0 (0) | 8 (7.0) | 32 (28.1) | 52 (45.6) | 22 (19.3) | 4 (3.0-4.0) |
| SBE are activities that mimic the reality of a clinical environment | 0 (0) | 2 (1.8) | 10 (8.8) | 61 (53.5) | 41 (36) | 4 (4.0-5.0) |

Abbreviations: SBE, simulation-based education; IQR, interquartile range.

Table presents participants attitudes toward SBE across multiple domains.

^a^ Values are presented as n and (%) of participants responding within each category on a 5-point Likert scale ranging from 1 (“strongly disagree”) to 5 (“strongly agree”).

^b^ Median (IQR) values are shown for each statement.
