# Supplementary material for: Registered Dietitians' Knowledge, Attitudes and Use of Simulation‐Based Education in Ireland: A Mixed‐Methods Study
Source: J Hum Nutr Diet. 2026 Jul 29;39(4):e70326. doi: 10.1111/jhn.70326 (PMC13420780; doi:10.1111/jhn.70326)
Supplement: Supplementary file 2 — Supporting File 2 [file JHN-39-0-s001.docx]

| **Themes (deductive)**  ***Supplementary table 1:*** ***Themes, subthemes, and illustrative quotes from interviews exploring dietitians’ perspectives on SBE in pre-registration dietetic training in Ireland (n=15)*** | | |
| --- | --- | --- |
| **Theme** | **Subtheme** | **Example Quotes** |
| **Knowledge & Definition** | **Knowledge gap** | ‘I don't know whether that's considered simulation now or not’ (P1)  ‘I suppose I've always kind of known of it, and then just in the literature, and from talking to colleagues, but… I'm not versed, I've never done a simulation-based education qualification or specific training on it, so what I do is self-learned.’ (P12) |
|  | **SBE perceived as formal or technology-based** | ‘I would have thought it was something a bit more high-tech and, AI or something… something like that.’ (P10)  ‘I signed up for this session on simulation and thought oh, this'll be really interesting. I was expecting them to go into a spaceship or something.’ (P13)  ‘…These kind of specialized scenarios for students… maybe we thought that was simulation more than what we were doing…on a day-to-day basis in class.’ (P14) |
|  | **SBE used without naming it** | ‘I suppose, we've probably always been doing it, but never called it that until the more recent years, when all this terminology came along.’ (P7)  ‘The terminology threw me a bit when I first saw it, but I think the concept has always been there.’ (P13)  ‘I wouldn't have come across the term before that, to be honest. It was only… doing the survey and watching the video, it became clear that it's probably something that you do without knowing that you're doing it, really.’ (P4) |
|  | **Definition of SBE** | ‘It's that middle ground bit that's trying to take the textbook learning and marry it with real-world clinical environments. That bit in the middle.’ (P11)  ‘It's like any sort of scenario, a case, or you have a problem…or you have something that's from the real world. But in a safe context for students, to help them prepare...and you want to make it as comfortable and as safe… for students as is possible. So you don't want to put them in a scenario where people are just being awkward for the sake of being awkward.’ (P1)  ‘Yeah, …it's probably a large, large term. It's difficult to put a specific, definition on it…you're just trying to prepare a student, …for any situations that might occur when you're seeing an actual patient, …so they feel more confident because…they've been exposed to that experience already in a more controlled manner.’ (P4) |
| **Setting** | **Recognised role of SBE** | ‘I suppose it'll have a place, but it shouldn't delay students getting to see real people and then practicing with real people.’ (P13)  ‘I think it's a really good precursor to seeing patients.’ (P12) |
|  | **SBE has no role on placement** | ‘I don't want to use simulation with students, I want to, you know, do real-life stuff with them…for you to come out to [Hospital name] and then sit in a clinic room pretending to see a patient, rather than actually seeing one. I just don't see the benefit of it.’ (P10)  ‘Whilst they're here in the hospital, I do think they really want to maximize the opportunities of working with other healthcare professionals and working with patients. So, doing simulation scenarios, when they're here in the hospital, maybe doesn't feel like it would be the best use of time…I thought of it as probably more an academic tool, as opposed to a tool to be used on a practical placement.’ (P6)  ‘I think while you have actual patients, it's good to go and see them, …dietitians aren't going to cause a huge amount of harm to a patient, if it's not going the right way…the idea is that you're on placement, so you're supervised, so there would be somebody there to jump in.’ (P8) |
| **Participant’s experience of SBE in their own pre-registration training** | **Positive experience** | ‘Looking back, at the time, we were so glad we got to practice on people that we know are not vulnerable… and that was really helpful before we went out on placement.’ (P15) |
|  | **Negative experience** | ‘I would have found it quite awkward, and not realistic.’ (P10) |
|  | **Acknowledges benefits of SBE despite low enjoyment** | Oh, I hate it. I totally hate it. I think it is a really good thing to do, because I think you have to know what words you're going to use, and I think the first time you walk into a setting with a patient or anybody, if you have… a bit of an idea of how you're going to approach it, it's very helpful, but I have to say, I hate doing it. (P9)  ‘Very nervous. I was very, very nervous….I think it's very valuable.’ (P11)  ‘Yeah, it's awkward, I suppose, would be the thing…but definitely helpful in terms of, kind of, expanding your knowledge and the practical side of things. (P4) |
| **Barriers & Enablers** | **Barriers to implementation** | ‘Well, it's very time-consuming, so, I mean, the main issue really is staff, like, we only have a very small staff on the programme.’ (P1)  ‘Finding a private space that you're going to be able to do it. The second thing would be the workload.’ (P10)  ‘We're never given opportunities to understand or learn more about it or to do training in it.’ (P15)  ‘…time pressures are definitely a consideration in the clinical environment.’ (P12) |
|  | **Enablers** | ‘…if there were scenarios set up and ready to go, that it wasn't the dietitian who's training has to create them from scratch… I think those kind of resources would be brilliant, and would be more likely to be used.’ (P9)  ‘By streamlining as much as possible… so rather than us doing it all separately, that it is a multidisciplinary effort.’ (P1)  ‘I think for those who hate it, like me, I think it's…communicating about how valuable it actually is.’ (P9)  ‘I definitely think some guidance around, tasks that could be done and how they're done would be helpful to clinical dietitians.’ (P12)  ‘I also think the universities and researchers and academics have a responsibility when they are…I won't say expert in an area, but they definitely have as much knowledge as you can have, or certainly far more than the average…they do need to provide that knowledge and expertise and filter it out to people, and again, try to find a way to translate how that might work clinically.’ (P15)  ‘I think it can be definitely accommodated if it's… if it was more proactively pushed, I suppose, from the…from the university level.’ (P4) |
| **Advantages & Risks** | **Advantages** | ‘I think there's less pressure for students when they're trying to learn those skills, when it's not a real person…they're not put on the spot.’ (P11)  ‘It's probably one thing students find the hardest, is to gain that confidence on the wards and with patients, so practicing talking to patients, practicing talking to doctors, talking to nurses and stuff, if you get more comfortable with that before you go see an actual patient, it'll come easier for you on the ward.’ (P4)  ‘It can be very daunting, very intimidating, speaking to a real-life patient for the first time. So…it helped build my confidence, definitely.’ (P6) |
|  | **Risks** | ‘There's always the risk that something sounds scripted, you know, that if you've practiced it over and over again, that it sounds like you're going in, kind of robotically scripting it.’ (P10)  ‘To me, one of the values is it increases not only knowledge, but it increases confidence, for the students, and you don't want for it to have the opposite effect, and maybe if it's ill-prepared, it will have the opposite effect.’ (P12)  ‘…it could be misleading, maybe, to students if they felt really confident in a simulation scenario, and then they were really overwhelmed or shocked when they came to a…real-life setting…they could be led into a false sense of security, almost.’ (P6)  ‘I think it can be challenging for students…everybody's watching you, and there's definitely a vulnerability within the students in doing simulation.’ (P7) |
| **Themes (inductive)** | | |
| **Theme** | **Example Quote** | |
| **Interdisciplinary simulation** | ‘I really think, the more multidisciplinary, the more real life it is, the more students can learn using simulation, not just about their own competence and skills, but also learn to appreciate what other people's competence and skill set is.’ (P1)  ‘…I think they would help: One, us to understand each other's roles, but secondly, to create an understanding of what we do, an understanding of where we can perhaps cross roads, so we're more interdisciplinary working rather than multidisciplinary working.’ (P12)  ‘I think in college as well, you don't really understand how…other people in the MDT…the reasons why they would see a patient. I think it was good for other people to understand the role of dietitians as well. And yeah, …it was good, because it wasn't a real patient, but it felt very real. It felt like we were on a ward.’ (P8) | |
| **Awareness of SBE benefits** | ‘I think for those who hate it, like me, I think it's…communicating about how valuable it actually is…because it is valuable. I might not like it, but I think you see the value in it later.’ (P9)  ‘I think, to be honest, I probably wouldn't do it off my own back, I suppose, unless they're [participant referring to HEI’s] like, okay, this is useful for students to do at this stage…I wouldn't be against that…but I probably would need a little bit of guidance on that.’ (P3)  ‘…sometimes we need to be shown all of the benefits, and that actually, you know, outweigh that little bit of unsettled discomfort, initially, of having to change our ways, and maybe do things a bit differently.’ (P11) | |
| **Importance of preparation and pre-briefing** | ‘I do think it needs to be prepared properly beforehand, and you can't just throw everyone into the situation, because then I think it's likely to…to me, one of the values is it increases not only knowledge, but it increases confidence, for the students, and you don't want to for it to have the opposite effect, and maybe if it's ill-prepared, it will have the opposite effect.’ (P12)  ‘I think we've learned in dietetics that without, you know, enough pre-briefing and preparing students for simulation… there's a risk of… of failing. You know, that they're not sufficiently prepared, and maybe they're not getting the best learning out of it. So I think…with the right structures, I think it's always beneficial. But we need to keep students'… needs to the fore, around feeling prepared, and feeling safe in simulation, and all of that.’ (P14) | |
| **SBE as a complement to clinical experience** | ‘I think it's useful but I don't think you can use it completely…I would never like to see it used to replace the real-life experience of placement.’ (P1)  ‘It doesn't get the student to the end point. They have to see the real experiences in real life, because you can simulate an experience, but the challenges that come left field from actually seeing a real patient can never be reproduced…’ (P7)  ‘I suppose it'll have a place, but it shouldn't delay students getting to see real people and then practicing with real people.’ (P13) | |

*Initial coding carried out deductively based on the COM-B model. Inductive analysis used to identify further patterns beyond the COM-B model.*

*Themes and subthemes derived from thematic analysis of interviews with CORU-registered dietitians in Ireland (n=15).*
